# Priorities and Challenges in Methodology for Human Health Risk Assessment from Combined Exposure to Multiple Chemicals

**DOI:** 10.3390/toxics11050401

**Published:** 2023-04-23

**Authors:** Dimitra Nikolopoulou, Evangelia Ntzani, Katerina Kyriakopoulou, Christos Anagnostopoulos, Kyriaki Machera

**Affiliations:** 1 Laboratory of Toxicological Control of Pesticides, Scientific Directorate of Pesticides’ Control and Phytopharmacy, Benaki Phytopathological Institute, 8 St. Delta Street, Kifissia, 14561 Athens, Greece; 2 Department of Hygiene and Epidemiology, Faculty of Medicine, University of Ioannina, PC 45110 Ioannina, Greece; entzani@uoi.gr; 3 Laboratory of Environmental Control of Pesticides, Scientific Directorate of Pesticides’ Control and Phytopharmacy, Benaki Phytopathological Institute, 8 St. Delta Street, Kifissia, 14561 Athens, Greece; k.kyriakopoulou@bpi.gr; 4 Laboratory of Pesticides Residues, Scientific Directorate of Pesticides’ Control and Phytopharmacy, Benaki Phytopathological Institute, 8 St. Delta Street, Kifissia, 14561 Athens, Greece; c.anagnostopoulos@bpi.gr

**Keywords:** chemical mixture, human risk assessment, combined exposure, risk assessment methodology, cumulative risk

## Abstract

This paper reviews key elements in the assessment of human health effects from combined exposure to multiple chemicals taking into consideration current knowledge and challenges to identify areas where scientific advancement is mostly needed and proposes a decision-making scheme on the basis of existing methods and tools. The assumption of dose addition and estimation of the hazard index (HI) is considered as a starting point in component-based risk assessments. When, based on the generic HI approach, an unacceptable risk is identified, more specific risk assessment options may be implemented sequentially or in parallel depending on problem formulation, characteristics of the chemical group under assessment, exposure levels, data availability and resources. For prospective risk assessments, the reference point index/margin of exposure (RPI/MOET) (Option 1) or modified RPI/normalized MOET (mRPI/nMOET) (Option 2) approaches may be implemented focusing on the specific mixture effect. Relative potency factors (RPFs) may also be used in the RPI approach since a common uncertainty factor for each mixture component is introduced in the assessment. Increased specificity in the risk assessment may also be achieved when exposure of selected population groups is considered (Option 3/exposure). For retrospective risk assessments, human biomonitoring data available for vulnerable population groups (Option 3/susceptibility) may present more focused scenarios for consideration in human health risk management decisions. In data-poor situations, the option of using the mixture assessment factor (MAF) is proposed (Option 4), where an additional uncertainty factor is applied on each mixture component prior to estimating the HI. The magnitude of the MAF may be determined by the number of mixture components, their individual potencies and their proportions in the mixture, as previously reported. It is acknowledged that implementation of currently available methods and tools for human health risk assessment from combined exposure to multiple chemicals by risk assessors will be enhanced by ongoing scientific developments on new approach methodologies (NAMs), integrated approaches to testing and assessment (IATA), uncertainty analysis tools, data sharing platforms, risk assessment software as well as guideline development to meet legislative requirements.

## 1. Introduction

The development of scientific approaches for assessing human health effects from combined exposure to multiple chemicals has been recognised as a key research and policy priority by European and international authorities, agencies and organisations over the past couple of decades [1,2,3,4,5,6,7,8,9,10,11,12,13].

Humans are exposed to a variety of chemicals that appear in mixtures, and, therefore, human health risk assessment to chemical mixtures is more relevant in describing the real-life human health concerns. Mixtures are defined as any combination of two or more chemical substances regardless of source or spatial or temporal proximity [14]. A more recent definition of chemical mixtures includes any set of chemicals to which an organism may be jointly exposed, and which may potentially cause an adverse combination effect, regardless of sources and exposure routes [12]. Three main categories of mixtures are identified describing how they originated: intentional mixtures that are manufactured and regulated as such (e.g., pesticide formulations), generated mixtures that originate simultaneously from a single source as byproducts of specific processes (e.g., drinking water disinfection, fuel combustion) and coincidental mixtures that originate from different sources and, although composed of unrelated chemicals, they may reach the same receptor population through a single medium (e.g., chemicals in groundwater) or through multiple pathways [15,16]. Coincidental mixtures may arise whenever chemicals are used on their own or as part of intentional mixtures or other complex products and thus their compositions may be unknown or variable over time. The term unintentional mixtures is also used, but there is no consistency on its definition. Kienzler and colleagues use the term unintentional mixtures to describe mixtures from one source generated by discharge during production, transport, use or disposal of goods (similar to generated mixtures) [17]. According to others, unintentional mixtures include coincidentally formed and variable mixtures originating from one or several sources, such as surface water contaminations or pesticide residues in food [18]. For the purposes of this review, we follow the definition coined by Bopp et al. [18]. Moreover, mixtures may also be classified as simple, having a known composition of up to ten chemicals, or complex, with compositions not fully known but including tens, hundreds or thousands of chemicals [15,19]. In comparison to other mixtures, it is possible to also identify similar mixtures when they have most or all chemicals in common with similar proportions [16,19]. More specific criteria for similarity assessments are described for regulated substances and products and are dependent on the regulatory context [20,21,22,23].

Current risk assessment practices are focused on single chemicals or intentional mixtures, as described in respective guidelines and legislation [24,25,26]. Human health risk assessment from exposure to unintentional mixtures is more challenging and highly depends on the complexity of these mixtures.

The necessity to perform human health risk assessment from combined exposure to multiple chemicals is not only highlighted since co-exposure to chemical mixtures occurs in everyday life but even more so because exposure to these mixtures may be associated with increased health concerns related to additive, synergistic, modifying or moderating effects as documented from in vitro, in vivo and human epidemiology data. Increased toxicity has been observed in receptor-based bioassays performed on selected chemical mixtures as they may occur in fresh waters [27]. Equipotent binary and ternary mixtures of the hepatotoxic pesticides imazalil, thiacloprid and clothianidin showed concentration-related increases in triglyceride accumulation in comparison to the effect of single chemicals in human hepatocarcinoma cell lines [28]. The combined effects of binary mixtures of cyproconazole, triadimefon or valproic acid on craniofacial malformations in rat whole embryo cultures and in zebrafish embryos have also been reported [29]. Similarly, effects in head skeleton (M-PQ angle) of zebrafish embryos were enhanced in binary mixtures of triadimefon or flusilazole with TCDD, thiram, VPA, prochloraz, fenpropimorph, PFOS or endosulfan [30]. Increased severity of hepatotoxicity, evidenced as increased liver weight and centrilobular hepatocellular hypertrophy and cytoplasm degeneration, was observed in female Wistar rats after 28-day gavage administration of binary mixtures of three pesticides, i.e., imazalil, thiacloprid or clothianidin, in comparison to single chemical exposures [31]. Kortenkamp and colleagues have reviewed published data providing evidence that mixture effects in experimental animals may also be elicited at doses below the effect doses of their individual components [32].

Epidemiological data published between January 2010 and December 2013 are linking combined exposure to multiple metals to paediatric health outcomes, including urinary tract birth defects, effects on reproductive and endocrine system outcomes with a focus on Pd-Cd synergistic effects on hypothalamic–pituitary–adrenal function, cognitive and motor development and behaviour [33]. Adverse health outcomes in children may be a consequence of in utero exposures during gestation and/or due to hand-to-mouth activity, which is common in young children under the age of three [32,34,35]. Lack of knowledge on human exposure and/or toxic effects has been recognized as an uncertainty in these assessments.

In this review, we will attempt to identify key elements in the assessment of human health effects from combined exposure to multiple chemicals taking into consideration current knowledge and challenges, identify areas where scientific advancement is mostly needed and propose a decision making scheme on the basis of existing methods and tools.

## 2. Points of Priority and Challenges in Problem Formulation

Problem formulation has been widely accepted as the initial step in the risk assessment process, highlighting its importance. It is the step where a decision is undertaken on whether a combined exposure to multiple chemicals is actually required, and the relevant methodology is decided and should involve the stakeholder matrix and the public [10,19,36,37].

Scoping and planning activities are necessary to describe the issue/problem to be addressed under specific legal framework(s), to present the risk management options and to allow identification of any specific issues for the stakeholders or the community [10]. Preliminary considerations on hazard identification and characterisation and exposure assessment are needed and proceed in an iterative manner. The following areas are considered critical in problem formulation and are, therefore, discussed more thoroughly in sections to follow, i.e., co-exposure patterns, whole mixture and component-based approaches, availability of hazard and exposure data.

a. Identification of co-exposure patterns

A key issue in problem formulation that is related to mixtures, and it is also known as the “gatekeeper” step [38], is the likelihood that chemicals co-occur under the conditions of the assessment and thus co-exposure is expected. Otherwise, a combined exposure assessment may be considered redundant.

Co-exposure refers to the internal exposure to the chemical substances at the time scale relevant for the adverse effect [39]. Co-exposure might be obvious when chemical components co-exist in an external mixture at a specific point in time. This might be relevant for the components of a regulated product (e.g., plant protection product, biocidal product), for pesticide residues in a monitoring sample concerning a specific food item or for contaminants at a target site (e.g., groundwater metabolites and degradation products, in situ generated substances, fuel combustion products in air). Different population groups might be exposed to specific chemical mixtures as part of their work (e.g., industrial workers handling a group of chemicals) or residence (e.g., residing fields of intense crop protection or emission sources). Co-exposures of breastfeeding infants (0–24 months old) to chemical residues through maternal milk or in utero co-exposures to chemicals that cross the placenta may impact the health status of developing embryos, foetuses and/or infants and thus are under continuous scientific investigation [40,41,42].

Depending on the toxicokinetics of the chemicals, co-exposure is still possible when external exposures to several chemicals do not occur simultaneously as long as bioavailability data demonstrate that these chemicals may reach the target site in the body at the same time scale, which is relevant for the adverse effect under assessment. The internal dose that is biologically effective at the target organ or tissue is responsible for the first interaction with the molecular target, that is, the molecular initiating event of an adverse outcome pathway [43]. Thus, co-exposure could be regarded as a function of external exposure and bioavailability at the target site and depends on the adverse effect under assessment and the time-scale relevant for that effect.

Since bioavailability of a chemical depends on its toxicokinetic properties, including maximum plasma concentration and time needed for plasma concentration to reach maximum levels, target tissue concentration, elimination rate through metabolism and excretion as well as tissue distribution and potential bioaccumulation, this information should be available for the identification of co-exposure patterns. This is particularly relevant for the assessment of short-term or long-term adverse effects caused by external exposures over several weeks, months or years to various chemicals and may be less critical in the risk assessment of acute effects, where the risk assessor could presume with a high degree of certainty that co-exposure will be achieved for external exposures taking place within a day (e.g., residues in food consumed within 24 h).

Data and information on toxicokinetics for a chemical may be obtained using in vivo animal tests or in vitro assays conducted according to relevant validated experimental protocols [44,45,46], (Q)SAR models and expert systems, mathematical representations of biological processes in the body through physiologically-based kinetic (PBK) modelling [47,48,49,50], human volunteer studies, medical records and other scientifically sound methods [51] and approaches [52,53]. PBK models have become important tools that facilitate extrapolation of doses that elicit biological responses in cellular systems in vitro to exposure levels in vivo ((Q)IVIVE) in the context of minimising animal testing [47,54].

b. Decision on whole mixture or component-based approaches

Two broad approaches have been widely developed and implemented to assess mixture toxicity: the whole mixture approach and the component-based approach [16,19,37].

In the whole mixture approach, the mixture is treated as a single entity for the risk assessment, and it is more relevant in cases where the mixture composition is partly known or mixture identity is not possible to characterise, such as in environmental samples [16,37]. Under this generic concept of whole mixture, the ATSDR [19] have distinguished the mixture of concern approach and the sufficiently similar mixture approach that could be considered in mixture risk assessment depending on data availability. It is recognised that having exposure and health impact data on the actual mixture being evaluated may be rare and mostly possible when data are being collected because the mixture poses a health concern due to large-scale production or co-occurrence at source of exposure, as is the case in occupational exposure settings, maternal milk, regulated products, etc. Toxicological and exposure data for the mixture of concern are used in the risk assessment. Information on medical records or epidemiology data might be considered on a case-by-case basis (e.g., in site-specific risk assessment). Since mixture composition might change with time or exposure conditions (e.g., change in distance from source of release), it might be necessary to adopt input parameters and revise risk assessment accordingly.

In cases of insufficient data on the mixture of concern, the sufficiently similar mixture approach may be used for risk assessment when health effects data or health-based guidance values are available on a mixture having the same chemicals but in different proportions or having most, but not all, chemicals in common and in similar proportions [19]. Sufficiently similar mixtures are expected to have similar health effects (e.g., PCB mixtures are considered sufficiently similar for risk assessment purposes).

Since exposure and effect data may only be produced for a limited number of mixtures due to resource and time constraints, component-based approaches may be used instead when the group of chemicals or its components are clearly identified [36].

Component-based risk assessment may be performed on pre-defined mixtures, as is the case for regulated products when there are no toxicological data on the mixture of concern or on a sufficiently similar mixture. Potential health effects of the mixture can then be predicted using toxicological data on individual mixture components and their concentrations in the mixture and using calculation methods (e.g., for acute oral, dermal or inhalation toxicity) or concentration thresholds (e.g., minimum concentrations that would trigger classification of a mixture for a specific target organ toxicity) [8]. This approach is solely hazard-based, taking into consideration the intrinsic properties of mixture components and the theory of additivity, assuming that each mixture ingredient contributes to the overall toxicity of the mixture in proportion to its potency and concentration.

In component-based approaches, it might be necessary to identify a group of chemicals that will be considered in the assessment. Grouping may be based on pragmatic considerations, regulatory criteria and scientific criteria. Regulatory criteria for grouping of chemicals are driven by legislative requirements and are set by risk managers. These criteria are considered to identify preliminary assessment groups of chemicals that share a common regulatory domain [36]. In line with European legislation for pesticides [55], all pesticides that could potentially be present in food marketed in the EU should be assessed for their possible inclusion in a common assessment group.

Scientific criteria for grouping could be based on structural, physicochemical, metabolic, toxicokinetic, biological, toxicological and/or exposure considerations. All members of the group should fulfil the same criteria. The more conditions included for grouping, the more likely it will be to exclude chemicals that do not fulfil all these criteria. On the other hand, the less specific the grouping criteria, the more likely it will be to identify large assessment groups that would be difficult to manage in the context of mixture risk assessment. Grouping of chemicals is further discussed in Section 3 of this review.

To overcome the issue of data availability, integrated approaches for risk assessment and uncertainty evaluation, including both a component-based approach and whole mixture approach, have also been proposed for environmental pollutants [11,56].

c. Data availability and sources

Collection of the relevant hazard and exposure data needed in order to perform human health risk assessment from combined exposure to multiple chemicals is not always an easy task due to legal, administrative and technical constraints and highly depends on data availability on individual mixture components.

Access to data may be subject to legal restrictions, as is the case with proprietary experimental toxicity and exposure data, such as data generated and owned by the industry for the approval of a pesticide or other regulated products in the European market under respective legislation [24,25], or the case of epidemiology data that fall under the EU mandatory rules of GDPR for use of personal data [57], including analytical results on biological fluids from monitoring studies. Substantial efforts have been undertaken towards data sharing in the context of chemicals registrations, with special reference to prohibition in repeating tests on vertebrate animals [58,59,60]. The Transparency Regulation ensures open access to scientific outputs in the EU food safety area [61]. These legal actions are examples of implementation of the 3R principles for more ethical use of experimental animals through Replacement, when possible, by non-animal methods, Reduction in their numbers and Refinement of experimental techniques so that animal experimentation is efficient [62]. The Chemical Strategy for Sustainability [12] has introduced the ‘one substance, one assessment’ approach that aims to overcome the technical and administrative obstacles in accessing data according to the principles that data should be easily Findable, Accessible, Interoperable and Reusable, referred to as the FAIR principles [63].

Careful selection of reliable and comprehensive data sources and tools is needed to collect relevant hazard and exposure data on individual mixture components in order to implement a proposed mixture risk assessment methodology. The risk of bias in experimentally derived data and the applicability and predictivity of model-predicted data from different sources should be considered to minimise uncertainty in the risk assessment process [64]. The online open access resources eChemPortal and INCHEM are broad directories that may be used as reference points for the collection of hazard data, as also recommended by WHO [65]. The eChemPortal [66] provides hazard information on a wide range of chemicals through access to European and international open access data sources, including the EFSA Open Food Tox [67], the ECHA REACH [68], the US EPA CompTox Chemicals Dashboard [69] and the IPCS INCHEM [70]. In the “Combined Exposures” database in eChemPortal last updated in April 2022, a collection of case studies implemented by the WHO, EFSA and USEPA on risk assessment of combined exposures to multiple chemicals is included. These case studies focus on grouping of chemicals for risk assessment on one or a few human health or environmental endpoint(s) [71]. The INCHEM portal itself provides access to internationally peer-reviewed information on chemicals from different exposure situations (environment, food, occupational) published through the International Programme on Chemical Safety (IPCS) and includes, among others, evaluations and monographs from the International Agency for Research on Cancer (IARC), the Joint Meeting on Pesticide Residues (JMPR), the Joint Expert Committee on Food Additives (JECFA), the Environmental Health Criteria (EHC) of the European Agency for Safety and Health at Work and many others [70].

The Information Platform for Chemical Monitoring (IPCHEM) is the reference access point of the European Commission for searching, accessing and retrieving chemical occurrence data collected and managed in Europe [72]. The IPCHEM may include data on environmental monitoring, human biomonitoring, food and feed products and indoor air from different European agencies, member state bodies, research centres and other projects and networks.

Availability of relevant hazard and exposure data may be higher for regulated chemicals for which data generation is mandatory for their registration and use in the European market, such as pesticides, or less comprehensive for non-regulated chemicals, such as mycotoxins and contaminants. Even for regulated chemicals, evidence on hazard does not always include toxicological properties of chemicals at all levels of biological organisation, including molecular, cellular, organ and whole organism. It is often the case that a hazard has been identified and characterised at organ or system level only. The implementation of a structured weight of evidence approach would be needed to assemble, weigh and integrate the available lines of evidence on toxicity [73,74,75].

The format of the available hazard and exposure data may also present a challenge in data collection and use. A great deal has been achieved so far in collection of publicly available hazard and exposure data, and efforts are undertaken in data sharing of proprietary information under specific collaborations, agreements and other research frameworks across legislations. However, further improvement on data sharing of proprietary information would further assist covering existing data gaps and uncertainties. Valorisation of existing data and methodologies is equally important as it is to produce new data and develop new methodological approaches.

## 3. Points of Priority in Grouping of Chemicals

A main step in component-based risk assessment from combined exposure to multiple chemicals is to identify the group of chemicals under assessment. This section will focus on consideration of specific scientific criteria for grouping of chemicals, prioritising chemicals within a group and prioritising assessment groups for the timely action on mixtures of concern with the purpose to protect human health.

a. Grouping based on hazard

For the combined risk assessment from exposure to chemicals, the scientific principles for grouping based on hazard are conceptually similar among different regulatory bodies, although terminology might be different. According to US EPA [76], mechanistic information and consideration of adverse outcome pathways (AOPs) are key in grouping pesticides. Common mechanism groups (CMGs) include two or more pesticide chemicals or other substances that cause a common toxic effect(s) by the same, or essentially the same, sequence of major biochemical events interpreted as mode of action (MOA) [77]. No common mechanism of toxicity could be established for thiocarbamates and dithiocarbamates. Neuropathology has been selected as the endpoint for use in a screening-level cumulative risk assessment of thiocarbamates to ensure that risks would not be underestimated [78]. Sufficient MOA/AOP information was available to establish distinct CMGs for organophosphates, N-methyl carbamates, chloracetanilides, triazines and pyrethrins/pyrethroids. Candidate CMGs may be identified in case of insufficient toxicity data when a common toxic effect by a common mechanism may be presumed based on selected criteria, such as structural similarity, mechanism of pesticidal action, general mechanism of mammalian toxicity or a particular toxic effect [79]. The candidate CMG is used to set a MOA/AOP hypothesis, estimate relative potency factors for the common toxic effect and proceed with grouping and risk assessment as with CMGs, underlying the additional uncertainty due to missing data [6].

According to the EFSA approach proposed in 2008 [80], cumulative assessment groups (CAGs) of pesticides are established on the basis of their common toxicological effects but not necessarily common mode of action. The establishment of relevant CAGs is considered the starting point for all cumulative risk assessments and thus of utmost importance. EFSA has considered as the basis for the establishment of CAGs the Danish Technical University (DTU) technical report [81], where a tiered approach for grouping had been proposed, including four levels with increasing level of refinement. In the EFSA Scientific Opinion [82], substances were grouped together at the following four CAG levels:

CAG level 1: organ/organ systems level;
CAG level 2: refinement on the phenomenological effect level;
CAG level 3: refinement based on mode of action;
CAG level 4: refinement based on mechanism of action.

**Figure 1:**
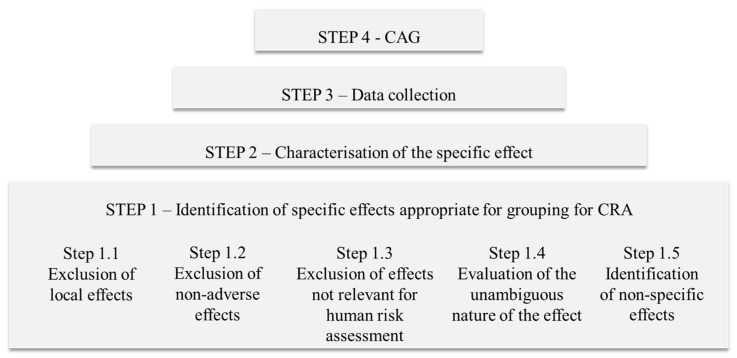
Grouping methodology developed by EFSA [82]. CAG: cumulative assessment group, CRA: cumulative risk assessment.

Allocation of a compound into CAG level 1 is based on an initial screening. As one compound may have an effect on several organs/organ systems, it may be included in more than one CAGs. Refinement is achieved on the basis of information on a specific phenomenological effect on the target organ/organ system in question (level 2) or on the mode/mechanism of action (CAG levels 3/4) pending data availability. In the EFSA Opinion [82], substances were grouped together based on the “occurrence of toxicologically relevant and unambiguously defined effects on the target organ i.e., on specific effects, even if the underlying initial biochemical events causing these effects have not (yet) been demonstrated experimentally”. The grouping methodology developed by EFSA in 2013 [82] included four key steps, as indicated in Figure 1.

In STEP 1, hazard identification was based on information analysis and expert judgement to determine the so called “specific effect”, a term used to describe the common effect among mixture components. In the following step (STEP 2), “indicators” of specific effects were defined, that is, observations or endpoints that characterise the specific effect (e.g., toxicity to the autonomic nervous system may manifest in form of different indicators, including miosis, salivation, lacrimation and urination). All relevant information would then be gathered (STEP 3) to form the CAG (STEP 4). Depending on the level of detail available for the specific effect, subgrouping may also be possible for different CAG levels.

This grouping methodology proposed by EFSA has been implemented for the development of CAGs for pesticide active substances with functional alterations of the central and peripheral nervous system [82,83], brain and/or erythrocyte acetylcholinesterase inhibition [84], chronic effects on the thyroid [82,85] and craniofacial alterations in the developing foetus [86] (Table 1).

Higher uncertainty is imposed when grouping is solely based on phenomenological effects or target organ toxicity due to inclusion of potentially less relevant substances (CAG Levels 1 and 2). Increasing specificity and lowering this uncertainty are achieved when mechanistic information on toxicity (MOA or AOP) is considered and applying a weight of evidence (WoE) approach [73]. This high level of knowledge and data on toxicity is expected in data-rich substances and constitutes the “gold-standard” of the hazard-based grouping approach since it allows for reduced uncertainty in chemical grouping [36].

In case of incomplete mechanistic information, data on structural similarity, common metabolites and toxicokinetic parameters may be used to avoid excluding chemicals from an assessment group that could lead to underestimation of the risk. Structural similarity can be assessed by describing chemical features, functional groups or precursor and breakdown products, qualitatively or quantitatively estimating the level of variation in each property [87]. Structural similarity assessments can be performed using software tools such as the OECD QSAR Toolbox [88], in silico models predicting toxicological properties or identifying potential molecular initiating events (MIEs). Molecular docking and machine learning tools can also be used for the prediction of MIEs and development of predictive in silico models.

Further identification of additional CAGs for effects on liver, the nervous system, reproduction and development may be triggered by the DTU assessment on specific identified effects and their related endpoints [89]. As part of this assessment, DTU has collected hazard data on pesticides and identified 257 substances with reproductive and developmental effects, 67 neurotoxic substances and 244 substances causing effects on the liver and biliary system, including the gallbladder. These pesticide groups can be used as the basis for the establishment of CAGs using pre-defined criteria as needed.

One of the main challenges in the implementation of the described grouping methodologies is data availability on single chemical hazard, dose responses, AOP and/or MOA data. It is anticipated that further development and implementation of new approach methodologies (NAMs), including in vitro and in silico methods, toxicokinetic modelling, read-across approaches, omics (transcriptomics, proteomics, metabolomics) techniques as well as improvement in data availability and data sharing practices for better use of all available data, will contribute further to the identification of molecular initiating events (MIEs), key events (KEs) and quantification of MIE/KE and KE/KE relationships. In addition, integration of data and tools under frameworks of integrated approaches to testing and assessment (IATA) will greatly facilitate filling knowledge gaps.

b. Prioritisation in chemical mixtures

Prioritisation of chemical mixtures for human health risk assessment involves the identification of mixtures of highest concern so that immediate and effective action can be taken to minimise health risks [90]. The criteria that have been considered for prioritising mixtures focus on hazard, exposure and risk of chemicals within mixtures.

According to FAO/WHO, in dietary risk assessment of chemical mixtures, priority should be given to compounds for which the estimated dietary exposure exceeds by “more than 10% the relevant health-based guidance value (HBGV) or in the absence of a HBGV, the calculated margin of exposure (MoE) is less than 10-fold of the MoE considered adequate for such a compound for at least one population” [91].

EFSA has considered the FAO/WHO approach and has further developed a risk-based prioritisation methodology that aims to identify low-priority chemicals that have marginal contribution to the cumulative risk based on specific cutoff values and may, therefore, be excluded from an assessment group without compromising the level of human health protection [36]. The cut-off values used to define low-priority chemicals depend on the context of the assessment and the prioritisation method selected, which can be combined risk-based approaches, single risk-based approaches and exposure-driven approaches. The identification of organ systems with high priority for mixture risk assessment is another critical aspect in the EFSA prioritisation methodology [92].

Priority for human health risk assessment could also be given to mixtures of priority substances. The US Agency for Toxic Substances and Disease Registry (ATSDR) has created a priority list of substances based on a combination of their frequency, toxicity and potential for human exposure at sites of national priority due to known releases or threatened releases of hazardous substances, pollutants or contaminants [93]. The China Ministry of Environmental Protection (MEP) has published a Prioritized List of Substances covering chemicals that are hazardous, persistent and may pose greater risk to the environment and human health [94], recently updated with additional substances including perfluorooctanoic acid (PFOA) [95]. The ECHA prioritises substances based on information on their intrinsic properties, their uses and production volumes in the EU market within the scope of authorisation requirements. The ECHA Candidate List of substances of very high concern contains 211 chemicals (January 2021) that may harm people or the environment [96].

Other reported criteria for the identification of priority mixtures include the presence of chemicals at levels close to their effect doses or chemicals that act with similar adverse outcome pathways or that have no threshold, or they are persistent to the environment [90]. The development of a harmonised methodology across scientific and regulatory bodies for the identification of priority chemicals and mixtures could potentially contribute to the timely control of human health risks.

## 4. Points of Priority and Challenges in Hazard Identification and Characterisation: Dose Addition, Independent Action, Interaction

Dose addition applies for similarly acting chemicals. It is based on the assumption that, when mixture components interact with the same target site of an organism, the total dose at the target site is increased and may lead to increased toxic effects of mixtures [97]. In this case, toxic effects of the different mixture components can be added after they have been scaled for their potencies. An index chemical is identified for which substantial information is available on the specific toxic action and all other mixture components are simply dilutions or concentrations of the index chemical. The index chemical could be the most data-rich chemical but not necessarily the most potent. The dose addition concept provides the scientific explanation of what has been called the “something from nothing” phenomenon, where chemicals at doses not eliciting measurable responses produce joint effects in combination [98]. Dose addition is the basis for the toxic equivalency approach followed for the assessment of dioxin-like compounds. The relative potency of dioxin-like compounds is estimated by comparison to 2,3,7,8-tetrachlorodibenzo-p-dioxin (TCDD) and calculation of the toxic equivalency factors [99]. Dose addition is also considered relevant for risk assessment of pesticide residues at the levels occurring in food. In fact, the EFSA Panel on Plant Protection Products and their Residues [100] has recognised that information justifying deviation from dose addition might be necessary, especially when these arguments are considered as a criterion for excluding substances from an assessment group. A proof of principle emerged from the EuroMix project, where experimental evidence was provided for the relevance and applicability of dose addition at concentrations occurring in food, i.e., at low exposure levels, regardless of similar or dissimilar modes of action [39,54].

Independent action is based on the assumption that components of the mixture do not interact at the biological target site and, therefore, do not influence each other’s toxicity. In this case, the risk from exposure to individual chemicals is sufficient and risk assessment from combined exposure to multiple chemicals is not needed.

When in mixtures, chemicals may influence each other’s uptake, metabolism, excretion and toxicodynamics, thereby modifying the magnitude and/or the nature of the toxic effect to more than additive (potentiation, synergism) or less than additive (antagonism) [90]. The members of the conazole fungicide family function through inhibition of the ergosterol synthesis pathway in fungi cell membranes, increase in cell membrane permeability and ultimately cell lysis and death. Each conazole is both metabolised by and affects the function of specific cytochrome P450 isoenzyme(s) and may affect the metabolism of other P450 metabolised substrates. The azole fungicide fluconazole is an inhibitor of CYP2C9 that is the key enzyme in the detoxification process of the anticoagulant warfarin, and, therefore, the presence of fluconazole inhibits warfarin metabolism and impacts its elimination process [101,102]. Inhibition of the metabolic enzyme CYP3A4 by the fungicide fludioxonil compromises the metabolism of difenoconazole, a substrate of CYP3A4, when in mixture experiments [103].

Certain persistent organic pollutants, such as polychlorinated dibenzo-p-dioxins (PCDDs), dibenzofurans (PCDFs) and biphenyls (PCBs) or polychlorinated naphthalenes, are slowly eliminated from the body, and repeated environmental exposure may lead to bioaccumulation, increasing the potential of co-exposure to other chemicals [104,105,106]. Polychlorinated naphthalenes are fast activators of phase I enzyme CYP1A1 in rats [107,108] and could potentially increase the turnover of certain chemicals produced in the body or xenobiotics.

Tissue residue analysis revealed alterations in the levels of pesticide residues in liver and kidneys in female Wistar rats after 28-day gavage exposure to binary mixtures of imazalil, thiacloprid and clothianidin in comparison to exposures to the single chemicals, suggesting possible toxicokinetic interactions [31].

Therefore, under certain circumstances, it is possible to have interactions of chemicals resulting in effects more or less than additive. The likelihood for interactions to occur between components in a chemical mixture highly depends not only on the intrinsic properties of the chemicals under consideration but also on the concentrations that these chemicals elicit toxic responses and how these correlate to co-exposure concentrations. Altenburger, et al. [97] have noted that there are no models available to estimate the biological response of components that interact and, therefore, influence the toxicity of each other. Boobis et al., have concluded that, at low doses (e.g., pesticide residues in food and feed), the magnitude of synergy does not exceed the levels predicted by additive models by more than a factor of 4 [109].

Investigation of human health effects from exposure to real-life mixtures may provide further insight on how to address mixture toxicity. Biomonitoring data from the Swedish Environmental Longitudinal, Mother and child, Asthma and allergy (SELMA) pregnancy cohort have indicated that human exposure to a mixture of endocrine-disrupting chemicals (MIX N) is associated with language delay in children. Toxicity testing has revealed that MIX N may interfere with hormonal pathways in human brain organoids and may be linked to neurodevelopmental effects through thyroid disruption in selected animal models at levels above a specific point of departure. A proportion of SELMA children had exposures above the levels of toxicological concern [110]. This study further highlights the need to integrate epidemiological data with experimental toxicology in mixture risk assessment frameworks.

## 5. Points of Priority and Challenges in Risk Assessment Methodology

**Figure 2:**
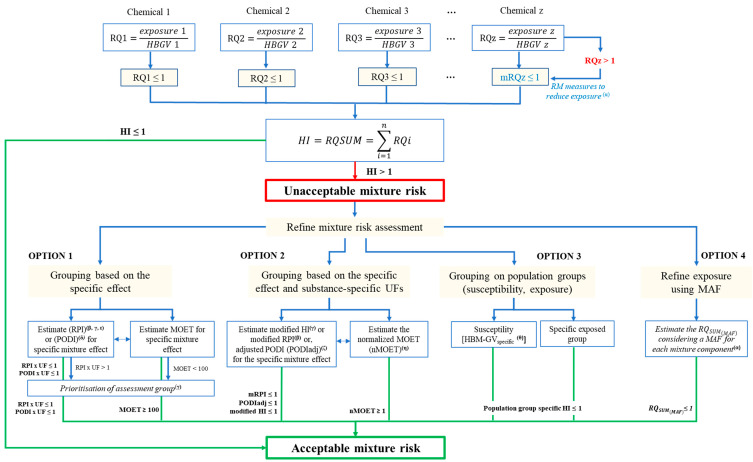
Proposed scheme for the assessment of human health risk from combined exposure to multiple chemicals based on currently available methodologies and options. HBGV: Health-Based Guidance Value, RQ = Risk Quotient, RQi: Risk Quotient for individual mixture components, mRQ: mitigated Risk Quotient, RQSum: Risk Quotient for the mixture, *n* = number of mixture components, *i* = a mixture component, HI: Hazard Index, RPI: Reference Point Index, mRPI: modified Reference Point Index, MOET: Margin Of Exposure Total (combined), HBM-GVspecific: Human BioMonitoring—Guidance Value for specific population group, MAF: Mixture Allocation Factor, RQSUM(MAF): Risk Quotient for the mixture adjusted using MAF. α: [111]; β: [112]; γ: [36]; δ: [37]; ε: [113], ζ: [114]; η: [115]; θ: [116].

One of the main challenges in the assessment of risk from combined exposure to multiple chemicals is considering all the available methodologies and implementing the most relevant one depending on the characteristics of the chemical group under assessment, data availability and resources. An attempt has been undertaken to include currently available methodologies and risk assessment approaches in a decision scheme for consideration in human health risk assessment of chemical mixtures (Figure 2).

Currently available methodologies and options on human health risk assessment from combined exposure to multiple chemicals may often appear independent to each other. When placed in a flow chart, it is shown that these methodologies are in fact complementary to each other and can be considered sequentially or in parallel to reach a conclusion on whether exposure to a specific chemical mixture is acceptable or not and under which conditions.

In a hypothetical priority group of chemicals (chemicals 1, 2, 3, …, z), dose addition is considered the default assumption, as previously discussed for chemicals interacting with the same target site of an organism (refer to Section 4). As a first step, the ratio of the estimated human exposure to a single chemical to the health-based guidance value (HBGV) for that chemical, known as the hazard quotient (*HQ*) [37] or risk quotient (*RQ*), is estimated (Equation (1)).

(1) $$HQ=RQ=\frac{exposure\;}{HBGV}$$

Human exposures may be externally calculated using appropriate prediction models and tools (e.g., the pesticide residue intake model, PRIMo) in prospective risk assessments or obtained through collection of data on chemical residues in food or the environment, including workplaces, in retrospective risk assessments. Internal human exposure levels may be retrieved from human biomonitoring (HBM) data or calculated from external exposures using physiologically based toxicokinetic (PBTK) computer modelling, such as the HTTK model [117] and RAIDAR model [118].

The levels of acute or chronic exposures for a given chemical without appreciable human health risk comprise the HBGV [119]. Thus, for dietary exposure assessments, the HBGV may be the ADI, ARfD or TDI estimated from toxicity studies using an appropriate point of departure (POD) or reference point (RP), such as the NOAEL, LOAEL, BMDL and considering uncertainty factors to account for interspecies, intraspecies variability and other uncertainties related to the quality of the data, LOAEL to NOAEL extrapolation, duration of exposure, etc. For non-dietary exposure assessments of professional and amateur users, bystanders, workers and residents, the HBGV may be the acceptable operator exposure level (AOEL) or the acute AOEL for short-term or acute exposures, respectively. The (A)AOEL is estimated as above with an additional correction for bioavailability of the substance based on oral absorption data. In case of retrospective risk assessments, in the estimation of the HQ, it is also possible to consider using the human biomonitoring guidance value (HBM-GV) or HBM-I derived from human biomonitoring data for internal substance concentrations at and below which there is no evidence of adverse health effects in human population [116,120]. The HBM-GV may be used in Equation (1) after being converted to the external equivalent concentration using PBTK computer modelling [121]. Alternatively, Equation (1) may be modified so that internal values are used, exposure refers to plasma or urinary concentration of a substance (parent chemical or metabolite) and the *HQ* is expressed as per unit *HBM-GV* of that substance:

(2) $$HQinternal=RQinternal=\frac{concentration\;in\;blood\;or\;urine\;i\;}{HBG-GV\;i}$$

Different options of utilising HBM data in risk assessment of chemical mixtures are being explored and reported in the literature [116,122]. In data-poor situations (e.g., certain metabolites or degradation products), the threshold of toxicological concern (TTC) and internal TTC (iTTC) can be used as toxicity metrices. The TTC approach is based on the assumption that human intake or exposure below certain thresholds do not elicit toxic responses [123]. Substances are grouped according to Cramer classification (classes I, II and III). In case substances have the potential to be DNA-reactive mutagens and/or carcinogens or belong to the groups of organophosphates or carbamates, specific TTC values are assigned. This concept is gaining interest in the scientific and regulatory community and used in various areas for chemical risk assessment (e.g., food contact materials, food flavouring agents, impurities in pharmaceutical products). However, additional work would be needed on the format, population, review and impact assessment of the existing databases that support the TTC approach towards broader regulatory acceptance [124,125]. The iTTC takes into account the specific toxicokinetics of a chemical and uses PBTK modelling to estimate internal doses that correspond to the TTC [126].

An acceptable risk from exposure to a single chemical may only be concluded when its HQ or RQ is equal to or lower than the value of 1, suggesting that exposure levels do not exceed the HBGV. When exposure levels to a single chemical are excessive and RQ >1 (as is the case for chemical z in Figure 1), then the risk to human health is not acceptable and immediate action should be taken to minimize that risk. The contribution of that chemical to the mixture risk is meaningful only after implementation of adequate risk management options that would lower human exposure levels up to the HBGV, resulting in a mitigated risk quotient (mRQ) of up to 1.

As a second step in the risk assessment process, the sum of the individual HQs or RQs for all components of the mixture, known as the hazard index (HI) [37] or RQSum [111], is estimated (Equation (3)). An acceptable mixture risk may be identified when *HI* ≤ 1 (equivalent to *RQSum* ≤ 1).

(3) $$I=\overset{n}{\underset{i=1}{\sum }}HQi\;or\;{RQ}_{SUM}=\overset{n}{\underset{i=1}{\sum }}RQi$$

where $Qi=RQi=\frac{Exposure\;i\;}{HBGV\;i}$.

Currently available risk assessment options are described below for consideration when the HI (or RQ_SUM_) exceeds the value of 1. All four options are implemented at the individual mixture component level. Options 1, 2 and 3 focus on the specific effect, i.e., on the common effect among mixture components. Options 1 and 2 are relevant for prospective risk assessments, whereas Option 3 is also relevant for retrospective risk assessments. Option 4 is based on protection goals for human health.

In Option 1, the reference point index (*RPI*) is estimated as the sum of the ratios of exposure to the reference point (*RPi*) for the specific mixture effect (Equation (4)) [112].

(4) $$RPI=\overset{n}{\underset{i=0}{\sum }}\frac{Exposure\;i}{RPi\;}$$

As an advantage towards the HI approach, where the HBGV of each substance is based on the critical effect of that substance but not necessarily on the common effect, thereby potentially overestimating the risk, in the RPI approach, the RP for the common effect is used. The RP is synonymous to the point of departure (POD), and it is the NOAEL, LOAEL or BMD for that effect [36]. The RPI is synonymous to the point of departure index (PODI) [37]. A safe use may be demonstrated when the RPI multiplied by an uncertainty factor is below or equal to 1 (RPI × UF ≤ 1). As a disadvantage towards the HI approach, where the HBGV of each substance is estimated from the critical RP or POD using substance-specific uncertainty factors, in the RPI approach, a common uncertainty factor for all mixture components, such as the factor of 100 for intra-/interspecies extrapolation, is considered, thereby potentially underestimating the mixture risk by disregarding chemical-specific uncertainties.

The RPI is also equivalent to the sum of the reciprocals of the margin of exposure (*MOE*) of each component or to the reciprocal of the total margin of exposure (*MOET*) (Equation (5)).

(5) $$\mathrm{RPI}=\overset{n}{\underset{i=0}{\sum }}\frac{1}{MOEi}=\frac{1}{MOET}$$

where $MOEi=\frac{RP\;i}{Exp\;i}$.

In the specific case that all mixture components share the same MOA/AOP based on detailed scientific information or at low exposure levels where a comparable dose–response curve may be presumed and dose addition clearly applies, then the RPI may be calculated using potency-adjusted exposure estimates, such as the relative potency factors (RPFs) or toxic equivalence factors (TEFs) [37]. The RPF of each component is defined as the ratio of the toxicity reference point of this component (RPi) by that of an index chemical (RP_index_) for which comprehensive toxicity data are available (Equation (6)) [37,127]. The TEF is a type of RPF where toxic equivalents of the index chemical are used in the estimation of potency-adjusted exposures. The most common example of this approach are dioxins, for which the TEFs are internationally established factors [99].

(6) $$RPFi=\frac{RP\;i}{RP\;index}\;or=\frac{POD\;i}{POD\;index}$$

The RPI is estimated by adjusting the exposure levels to each of the mixture components using their RPF values and Equation (1) is amended accordingly (Equation (7)) [113].

(7) $$RPI\;\left(common\;MOA/AOP\right)=\overset{n}{\underset{i=0}{\sum }}\frac{Exp\;i\;x\;RPF\;i}{RPindex}\;$$

When a safe use is not demonstrated using the RPI approach for the initial assessment group, it is possible to proceed with the refinement of the assessment group considering prioritization methods on the basis of combined and single-substance risk and exposure metrics [36]. The prioritized assessment group (i) may only include those chemicals that contribute by more than 10% (cutoff) to the combined risk, i.e., the risk drivers, (ii) may exclude low-priority chemicals when their individual hazard quotient or MOE falls below a pre-defined cutoff value, i.e., low-risk chemicals, or (iii) may exclude low-priority chemicals for which the probability of co-exposure is low. According to the prioritization approach, the RPI for the prioritized group of chemicals may be calculated using Equation (7).

In Option 2 illustrated in Figure 1, increasing the specificity of the assessment is possible by estimating a modified reference point index (mRPI) that is the sum of reference point quotients of the individual components (RPQi) estimated for the specific mixture effect. Each RPQi is expressed as a ratio of exposure to the RPi or PODi (NOAEL, LOAEL or BMDL) for the specific mixture effect and corrected by the appropriate compound-specific uncertainty factor (UFi) (Equation (8)) [112].

(8) $$mRPI=\sum_{i=0}^{n}RPQi$$

where $PQi=\frac{Exposure\;i\;*UF\;i}{RPi}$.

The mRPI approach has an advantage towards the RPI approach since a specific uncertainty factor (UF) derived for each reference point as recommended by available guidance to encounter for interspecies/intraspecies variability, completeness of the database, extrapolation for duration of exposure, etc. [128,129] is used and, therefore, reflects the uncertainty linked to each specific mixture component. However, it is not possible to apply potency scaling in Equation (8) when the UFi is different for each mixture component. The mRPI is synonymous to PODI_adj_ [114] and to effect-specific HI or modified HI (mHI) [36]. mRPI values below 1 indicate acceptable human health risk.

Instead of the mRPI, it is also possible to estimate a normalised MOET (nMOET) where the MOE of the individual mixture component is normalized using an acceptable MOE (AMOE) that may be compound-specific. Although the concept of nMOET was introduced [115] to overarch the risk across silos, including pesticides, persistent organic pollutants and food additives, it could still be relevant for addressing the risk of any mixture where a different AMOE is relevant for each component, thereby achieving higher specificity in risk assessment (Equation (9)). This may be of particular relevance for substances leading to the same adverse outcome via different mechanisms, as is the case for endocrine-disrupting chemicals acting through different modalities. An acceptable risk is identified when *nMOET* ≥ 1.

(9) $$\frac{1}{nMOET}=\frac{1}{\frac{MOE\;1}{AMOE\;1}}+\frac{1}{\frac{MOE\;2}{AMOE\;2}}+\frac{1}{\frac{MOE\;3}{AMOE\;3}}+\ldots +\frac{1}{\frac{MOE\;z}{AMOE\;z}}\;$$

The reciprocal of the *nMOET* is equivalent to the mRPI in case the AMOE reflects the UFi. However, the AMOE could be based on risk management options that entail certain protection goals not considered in the mRPI estimation. In that respect, the nMOET could even be treated as a higher-tier approach to be followed when the mRPI exceeds the value of one to describe the conditions under which an acceptable risk may be identified. A decision on whether an acceptable risk is identified may then be undertaken by assessing whether the AMOE offers an adequate level of human health protection.

In Option 3 illustrated in Figure 1, it is possible to increase specificity in hazard index estimation by focusing only on human health effects or exposure levels relevant to a selected population group of interest. Human health effects may be population-specific in retrospective risk assessments when human biomonitoring data are available and specific HBM-GV or HBM-I (HBM-GV_specific_) are derived for vulnerable population groups and/or for certain phases of life by considering differences in physiology (e.g., women of child-bearing age, children, elderly) or for occupationally exposed adults [116]. The HI for the specific population group is then estimated as shown in Equation (10). This approach narrows the relevance of the assessment to specific population groups and may not be specific to the common mixture effect depending on data availability. It may also assist decision making when mixture risk may only be acceptable to specific population groups, whereas further action would be needed for other population groups or the general population to minimize risk to human health.

(10) $$HI\mathrm{internal}\;\left(specific\;population\;group\right)=\overset{n}{\underset{i=1}{\sum }}\frac{Conc.\;in\;blood\;or\;urine\;i\;}{HBM\;-\;GVspecific\;i}\;$$

Exposure levels may be population-specific in both prospective and retrospective risk assessments when they are collected for different population classes (adults, toddlers, children) and/or countries.

In contrast to Options 1 to 3 focusing directly on hazard and/or exposure, in Option 4, an additional safety factor, the mixture assessment factor (MAF) [12] or mixture allocation factor or “risk cup” [111,130], is applied in the risk assessment of single chemicals with the purpose to generically cover for combined exposure without performing a mixture-specific assessment.

The magnitude of the MAF may be determined by the number of mixture components, their individual potencies and their proportions in the mixture. The MAF is applied for each chemical according to Equation (11) until the mixture risk becomes acceptable, i.e., the *RQ_SUM(MAF)_* = 1 (Equation (11)).

(11) $${RQ}_{SUM(MAF)}=\overset{n}{\underset{i=1}{\sum }}{RQ}_{MAF}\;$$

where ${RQ}_{MAF}\;i=\frac{RQ\;i\;}{MAF\;i}$.

In this process, the MAF may only affect a fraction of mixture components, those contributing more to the overall risk (risk drivers) for which the MAF would be greater than 1 and up to the number of mixture components, whereas, for others, the MAF would be equal to 1, i.e., RQ_MAF_ i = RQi. An indication of the number of mixture components that contribute to the overall risk may be provided by estimating the maximum cumulative ratio (MCR). By definition, the MCR reflects the contribution of the RQ of the chemical with the highest risk quotient (RQ_max_) in mixture HI i.e., MCR = HI/RQ_max_ [131] or MCR = RQ_SUM_/RQ_max_. An MCR close to 1 would suggest that a single risk driver is responsible for mixture toxicity, whereas, the higher the MCR, the more likely it is that more mixture components have high contributions [131,132].

In order to apply a sufficiently protective, but not overly conservative, MAF, KEMI has performed an impact assessment [111] showing that an MAF of 10 is sufficiently protective for mixtures of up to 30 chemicals, whereas, for mixtures with more than 30 components (*n* > 30), an MAF of roughly n/2 would be considered adequate. The highest MAF that may be used for all mixture components, reported also as MAF_ceiling_ [132], would be equal to the number of chemicals in the mixture, under the assumption of dose additivity and considering that all mixture components contribute equally to the mixture risk (equipotent). However, this value would be overprotective for most real-world mixtures.

Unlike other assessment factors [5,128], the MAF is driven by (co-)exposure considerations and protection goals and could be applied in cases where conclusive mixture risk assessments could not be routinely conducted due to significant knowledge and data gaps [12,133].

## 6. Conclusions and Future Considerations

Several approaches have been developed for the human health risk assessment from combined exposure to multiple chemicals. Currently available component-based risk assessment approaches are included in a decision scheme for consideration in human health risk assessment of chemical mixtures. The default assumption of dose addition is considered as a starting point. Although dose addition is scientifically relevant for low exposure concentrations, as is the case for dietary exposures to pesticide residues, it is currently unclear to what extent dose addition is scientifically valid for non-dietary risk assessments due to potential chemical interactions (potentiation, synergism) of mixture components at higher exposure levels. Differences in the shape of the dose–response curve between mixture components at higher exposure levels is an important criterion for the applicability of potency scaling under the dose addition approach. Further research would be needed on identification and quantification of chemical interactions at non-dietary exposure levels to avoid underestimating human health risk.

The proposed decision scheme is structured to consider the HI as a generic approach and proceed with increasing specificity in mixture risk assessment by focusing on the specific mixture effect, substance-specific uncertainty factors and priority population groups and substances, with the purpose to assess whether an acceptable mixture risk is possible and under which conditions. Data availability on toxicity of individual mixture component level, dose responses, AOP and/or MOA as well as human exposure data may present a challenge in mixture risk assessment. It is anticipated that further development and implementation of in vitro and in silico methods, toxicokinetic modelling, read-across approaches, omics (transcriptomics, proteomics, metabolomics) techniques as well as improvement in data availability and data sharing practices for better use of all available data and integration of data and tools under IATA frameworks will greatly facilitate filling knowledge gaps.

The development of a harmonised methodology across scientific and regulatory bodies for the identification of priority chemicals and mixtures could potentially contribute to the timely control of human health risks. Integrated approaches for human health risk assessment from combined exposures to multiple chemicals, including both component-based and whole-mixture aspects, and quantifying the underlying uncertainty would make better use of all available data and tools and provide options for decision making under a more holistic approach.

In data-poor situations, the MAF has been proposed as an available option. Criticism on the scientific grounds of a hazard-based MAF has triggered further research towards the development of an MAF methodology. Although the necessity to apply the MAF with the purpose to manage the risks of exposure to unintentional combinations of chemicals in the EU has been acknowledged by regulators, further research would be needed to scientifically support an MAF methodology and further data from monitoring, modelling and experimental studies to estimate the MAF magnitude [134,135,136,137]. The first case study performed on the use of human biomonitoring (HBM) data, harmonised within the European project HBM4EU to provide a better estimate of the MAF, has recently been published, covering a broad range of priority substance groups [132].

Several projects and activities are currently scheduled on mixture risk assessment under the European Partnership for the Assessment of Risks from Chemicals (PARC) [138] aiming to develop the next generation risk assessment, thereby efficiently protecting human health from exposure to chemicals towards a more sustainable environment.

## Figures and Tables

**Table 1:** Establishment of cumulative assessment groups of pesticides for their effects on the nervous system, the thyroid and the developing foetus.

Endpoint	CAG Level		Reference
	CAG Level 1 (Organ/ Organ System)	CAG Level 2( Refinement on Phenomenological Effect)	
Neurotoxicity (acute)	Pesticide active substances affecting the nervous system, either central or peripheral	Substances with different general targets concerning neurotoxicity: - Effects on motor division of the nervous system (*n* = 119) - Effects on sensory and autonomic function (*n* = 101) - Neurochemical effects (brain and/or erythrocyte acetylcholinesterase (AChE) inhibition) (*n* = 47) - Neuropathological changes in neural tissue (*n* = 19)	[82,83]
Neurotoxicity (chronic)	Pesticide active substances producing chronic effects on the nervous system	Substances with chronic erythrocyte acetylcholinesterase inhibition (*n* = 47)	[84]
Thyroid toxicity	All substances affecting the thyroid hormone system (gland or hormones)	Different general targets, including thyroid tissue and associated hormone systems: - Substances affecting the thyroid parafollicular cells (C-cells) and/or calcitonin system (CAG2A) - Substances affecting thyroid follicular cells and/or the thyroid hormone (T3/T4) system (CAG2B)	[82,85]
Craniofacial alterations (acute)	Pesticide active substances and their metabolites causing craniofacial alterations	- Substances (*n* = 39) causing alterations due to abnormal skeletal development (CAG-DAC) - Substances (*n* = 41) causing head soft tissue alterations and brain neural tube defects (CAG-DAH)	[86]

# includes effects on locomotor activity, muscle strength, coordination and equilibrium.

## Data Availability

All information sources are referenced. These might include data from publicly available databases or data published in research articles. Data are generated in this review in the form of proposed scheme for the assessment of human health risk from combined exposure to multiple chemicals based on currently available methodologies and options. There is no need for ethical consent in the information used.
